# Lowe Syndrome Protein OCRL1 Supports Maturation of Polarized Epithelial Cells

**DOI:** 10.1371/journal.pone.0024044

**Published:** 2011-08-25

**Authors:** Adam G. Grieve, Rachel D. Daniels, Elena Sanchez-Heras, Matthew J. Hayes, Stephen E. Moss, Karl Matter, Martin Lowe, Timothy P. Levine

**Affiliations:** 1 Department of Cell Biology, UCL Institute of Ophthalmology, London, United Kingdom; 2 Faculty of Life Sciences, University of Manchester, Manchester, United Kingdom; Georgia Health Sciences University, United States of America

## Abstract

Mutations in the inositol polyphosphate 5-phosphatase OCRL1 cause Lowe Syndrome, leading to cataracts, mental retardation and renal failure. We noted that cell types affected in Lowe Syndrome are highly polarized, and therefore we studied OCRL1 in epithelial cells as they mature from isolated individual cells into polarized sheets and cysts with extensive communication between neighbouring cells. We show that a proportion of OCRL1 targets intercellular junctions at the early stages of their formation, co-localizing both with adherens junctional components and with tight junctional components. Correlating with this distribution, OCRL1 forms complexes with junctional components α-catenin and zonula occludens (ZO)-1/2/3. Depletion of OCRL1 in epithelial cells growing as a sheet inhibits maturation; cells remain flat, fail to polarize apical markers and also show reduced proliferation. The effect on shape is reverted by re-expressed OCRL1 and requires the 5′-phosphatase domain, indicating that down-regulation of 5-phosphorylated inositides is necessary for epithelial development. The effect of OCRL1 in epithelial maturation is seen more strongly in 3-dimensional cultures, where epithelial cells lacking OCRL1 not only fail to form a central lumen, but also do not have the correct intracellular distribution of ZO-1, suggesting that OCRL1 functions early in the maturation of intercellular junctions when cells grow as cysts. A role of OCRL1 in junctions of polarized cells may explain the pattern of organs affected in Lowe Syndrome.

## Introduction

The intricate 3-dimensional architecture of organs relies on cells exchanging information with their neighbours through direct contact at intercellular junctions. Two main types of junction in epithelial cells (adherens junctions, and tight junctions) mediate cell polarization, allowing the formation of a specialized apical surface. The junctions have many components, including integral membrane proteins that bridge between cells to create a permeability barrier across the epithelium, and associated cytoplasmic proteins which form electron-dense plaques from where many aspects of cell function are regulated, including cell division, cell shape (largely via effects on the actin cytoskeleton), and membrane traffic [1]. Problems with junctional integrity can underlie a range of cellular pathologies, due to loss of the barrier or epithelial-mesenchymal transition as a prelude to cancer. When cells first contact each other, primordial junctions form, which contain components that subsequently are found both in adherens junctions (E-cadherin) and in tight junctions (Zonula occludens-1, ZO-1). In some cases, proteins ultimately destined for different junctions interact during junction formation, for example ZO-1 and α-catenin [2]. As the epithelium matures, cells change from cuboidal to columnar shape, and junctions mature by a poorly understood exchange of components that includes the separation of adherens and tight junction components [1].

Phosphoinositides (PIPs) have been shown to play important roles at intercellular junctions. Many junctional proteins interact directly with PIPs [3], and segregation of PIP_3_ from PI45P_2_ drives separation of apical and basolateral membrane compartments [4]. Enzymes that regulate PIPs may therefore be crucial in epithelial development. Relating to this, PI4P 5-kinases [5], [6] and a PI45P_2_ phospholipase [7] have been localized to, and function at, junctions. Out of the family of 10 enzymes in mammals that remove the 5-phosphate from PIPs, capable of converting PI45P_2_ back to PI4P, none has been found at junctions [8]. We are studying one of these enzymes: OCRL1. OCRL1 is mutated in patients with the Oculocerebrorenal disease of Lowe, also called Lowe Syndrome, which is dominated by congenital bilateral cataracts, severe mental retardation, and proximal renal tubulopathy, which progresses to renal failure.

OCRL1 and one other 5-phosphatase Inpp5b (Inositol polyphosphate 5-phosphatase) [9] form a 5-phosphatase sub-family defined by a unique domain structure, as their carboxy-termini contain paired ASH (ASPM, SPD-2, Hydin [10]) and Rho-GTPase activating protein (-GAP) domains, the latter lacking the critical residue for catalysis [11]. Previously, OCRL1 has been shown to regulate both membrane traffic from endosome-to-*trans*-Golgi network [12], and the actin cytoskeleton [13], [14], [15]. These effects are thought to be mediated by the interactions of OCRL1 with small GTPase regulators, including many Rabs, Arf1 and 6, Rac and Cdc42 [11], [16], [17], [18]. Indirectly, OCRL1 might affect the many peripheral plasma membrane proteins involved in endocytosis and actin polymerization that use PI45P_2_ as a co-receptor [19]. The presence in OCRL1 of binding sites for clathrin, the adaptor protein AP-2, the endocytic adaptor APPL1, and other endocytic proteins [20], [21] strengthens the link to endocytosis [22], [23], [24], which tends to support the proposition that the tubulopathy of Lowe Syndrome derives from altered trafficking of megalin [23], [24]. However, renal epithelial cells lacking OCRL1 directly tested for endocytic traffic of megalin showed no reduction [25], which indicates another mechanism should be considered.

To date, most investigations on OCRL1 have used fibroblasts (Cos-7, NRK or skin fibroblasts), or dedifferentiated epithelial cell lines (HeLa) [12], [13], [15], [23]. In contrast, the cell types most affected in Lowe Syndrome are highly polarized: renal proximal tubule and lens epithelium, neurons and glia. To reproduce the specialized cell biology of Lowe Syndrome, we have studied OCRL1 in polarized epithelial cells. In renal tubular and intestinal epithelial cell-derived lines we find that OCRL1 plays a role in development of polarized epithelial cells. In addition, there are potential direct links between OCRL1 and junctions, as a pool of OCRL1 localizes to junctions, and OCRL1 forms complexes with key junctional components including ZO-1 and α-catenin. These results provide a new insight into how loss of OCRL1 might specifically affect epithelial cells.

## Results

### OCRL1 localizes to junctions between early confluent MDCK and Caco-2 cells

**Figure 1:**
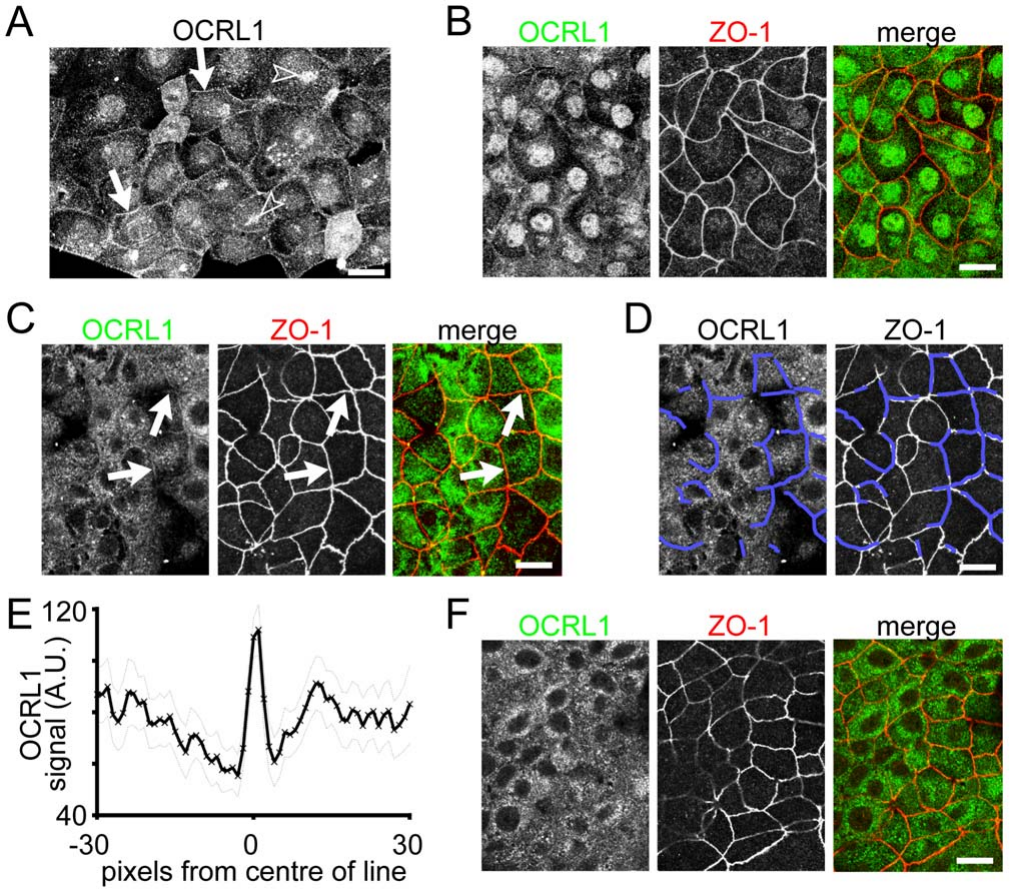
OCRL1 localizes to junctions between early confluent MDCK and Caco-2 cells. (**A and B**) MDCK cells were plated onto 35 mm tissue culture plastic dishes and allowed to grow for 16 hours achieving overall confluency of 40%. Cells were then fixed with 4% paraformaldehyde, permeabilized, blocked and processed for OCRL1 immunostaining and, in (B) only, ZO-1 co-staining, where the merge shows OCRL1 coloured green and ZO-1 in red. In A, there is faint targeting of OCRL1 to linear regions outlining cells (arrows), as well as bright peri-nuclear staining reminiscent of the Golgi (arrowheads) and faint nuclear staining. In (B), linear OCRL1 staining is seen to co-localize with ZO-1. (**C/D/E**) Caco-2 cells were grown and imaged as in B. Arrows in (C) indicate faint linear OCRL1 co-localizing to junctions. (D and E) show further analysis to confirm junctional targeting of OCRL1. In (D), linear regions where OCRL1 faintly co-localizes with ZO-1 are indicated by blue lines. In (E), lines 61 pixels long and 5 pixels wide have been drawn perpendicular to the blue lines (i.e. at junction and 30 pixels either side), and fluorescence scanned along their length using ImageJ (NIH). The graph shows the average OCRL1 fluorescence (arbitrary units  =  fluorescence brightness, scale up to 255). Thin dotted lines show ± s.e.m. (n = 29). Only the two points at the central peak showed significant differences (p = 0.0003 and p<0.002) from the remaining points. (**F**) Caco-2 cells were grown as in (A) but for 48 hours, achieving overall confluency of 70%, and then imaged as in (A). No junctional OCRL1 was seen under these conditions. Note the partial nuclear localization of OCRL1 in MDCK cells, which was not seen in Caco-2 cells. Scale bars are 20 µm.

In Madin-Darby canine kidney (MDCK) cells at an early stage of junction formation, there was significant, yet faint linear staining at the cell periphery (Figure 1A), which co-localized to some extent with ZO-1 (Figure 1B). To confirm that the junctional staining with anti-OCRL1 antibodies is specific, we repeated the assay in cells depleted of OCRL1 by RNA silencing, which showed that both the junctional staining and the internal (peri-Golgi) staining were completely dependent on the presence of OCRL1 (Figure S1).

To test whether junctional OCRL1 is a general phenomenon of polarized epithelial cells, we examined the human intestinal Caco-2 cell line. Caco-2 cell monolayers grown to confluence for short periods of time showed some OCRL1 localization at the periphery of cells, though this was to a lesser extent than in MDCK cells (Figure 1C/D/E). To demonstrate the subtle junctional targeting of OCRL1 (Figure 1C), junctional regions were analysed by line-scans. Firstly, we identified putative regions of junctional OCRL1 (Figure 1D), then lines were drawn perpendicular to the identified linear staining, and fluorescence along these lines was analysed as a function of distance from the peak of ZO-1 staining. This showed a significant rise in OCRL1 precisely at the intercellular junction (Figure 1E). This is not caused by cross-reaction of antibodies detecting OCRL1 with antibodies binding ZO-1, as single stained cells also showed junctional OCRL1 (compare Figure 1A and B).

When Caco-2 cells were grown for a further ≥24 hours, reaching greater confluency and allowing maturation of junctions, junctional OCRL1 was lost (Figure 1F). In comparison to polarizing epithelial cells, linear OCRL1 was never seen at the cell periphery in de-differentiated epithelial cells such as HeLa cells or COS-7 cells (data not shown and [12], [23]). Thus, peripherally enriched OCRL1 is seen only in epithelial cells, and only at the early stages of junction formation.

**Figure 2:**
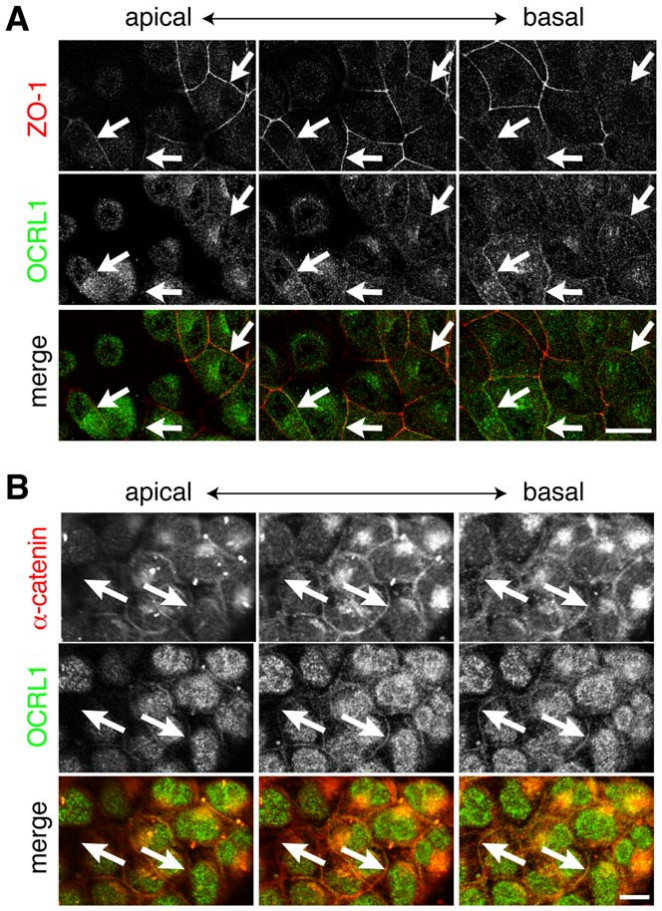
OCRL1 localization is similar to α-catenin, slightly basal to ZO-1. (**A**) OCRL1 and ZO-1 in MDCK cells, as in Figure 1B, showing multiple confocal sections for a single group of MDCK cells. Arrows indicate the same three sites in all images, where junctional ZO-1 is observed in the apical and middle section, but not the basal section, while OCRL1 is observed more strongly in the basal section than the other two. (**B**) OCRL1 and α-catenin (red in the merge) in MDCK cells, as in (A). Arrows show a typical junction where staining for both OCRL1 and α-catenin is maximal in the basal section. Scale bars are 10 µm.

By comparing adjacent optical sections in MDCK cells, we found that OCRL1 was slightly basal to ZO-1 (Figure 2A), and the same effect was also seen in Caco-2 cells (data not shown). To examine this further, we co-stained MDCK cells for OCRL1 and the adherens junction protein α-catenin, which localizes basal to ZO-1 in mature epithelia. There was significant colocalization between OCRL1 and α-catenin throughout the whole lateral compartment (Figure 2B). At these early stages when cells are not fully polarized, in addition to the classical junctional localization of α-catenin observed in mature epithelial cell monolayers, we observed a high level of intracellular α-catenin, as seen previously [26]. In summary, at an early stage of junction formation, a small proportion of OCRL1 targets intercellular junctions in multiple epithelial cell lines, being distributed more similarly to a marker of adherens junctions (α-catenin) than to a marker of tight junctions (ZO-1).

### Junctional localization is determined by the carboxy-terminus of OCRL1 and is conserved in Inpp5b

**Figure 3:**
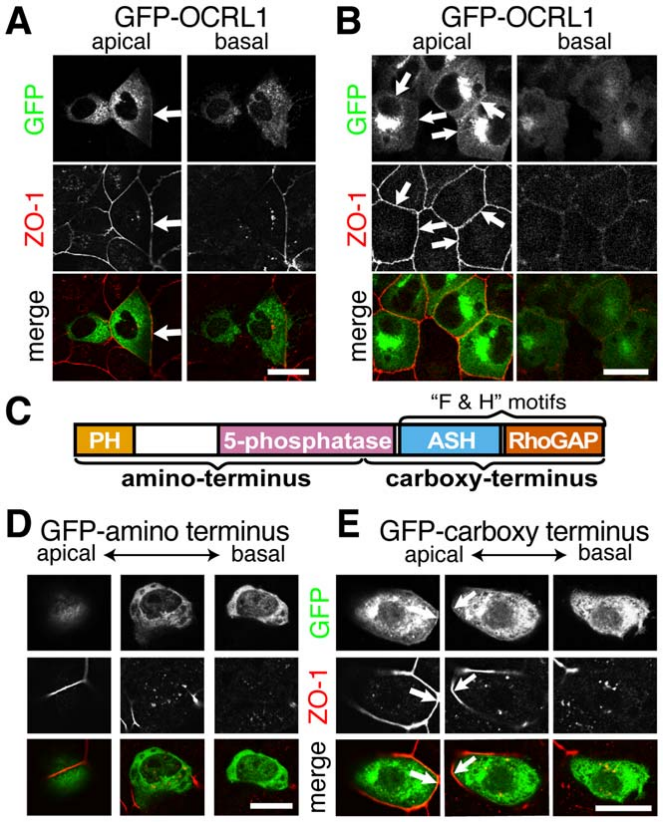
Junctional localization is determined by the carboxy-terminus of OCRL1 and is conserved in Inpp5b. GFP-tagged constructs were transfected into cells at low confluency: (**A, D, E & F**) Caco-2, (**B**) MDCK. (**C**) Domain structure of OCRL1, indicating constructs used in this figure, and the region of OCRL1 known to bind “F & H” motifs. Constructs used: (A & B) GFP-OCRL1, (**D**) GFP-OCRL1–amino-terminus, (**E**) GFP-OCRL1–carboxy terminus. 24 hours post-transformation, cells were fixed in methanol and processed for ZO-1 immunostaining as in Figure 1B. In all cases, apical and basal confocal sections are compared, with intermediate sections included where they add information. For full length OCRL1 (A and B), and for OCRL1-carboxy terminus (E), GFP-tagged constructs (top panels) partially colocalize (arrows) with apical junctions, as marked by ZO-1 (bottom panels), but there is no peripheral GFP in basal sections. By contrast, GFP-OCRL1-amino terminus (D) is not at apical junctions. Scale bars are 10 µm.

We next expressed GFP-tagged chimeras to examine how OCRL1 targeting is achieved. First, we found that GFP-tagged full-length OCRL1 targeted junctional regions close to junctional ZO-1 in both Caco-2 and MDCK cells (Figure 3A and B). To further map the determinants within OCRL1 responsible for junctional targeting, we expressed amino- and carboxy-terminal halves of OCRL1 tagged with GFP (Figure 3C). The amino-terminal half of OCRL1 was diffusely cytosolic, restricted to the cytoplasm (Figure 3D). In contrast, the carboxy-terminal portion of OCRL1 localized to apical junctions, as well as targeting to internal perinuclear membranes (Figure 3E). Expressing these constructs in non-polarized cells (HeLa) showed that the amino-terminus was again cytosolic, while the carboxy-terminus targeted perinuclear membranes similar to full-length OCRL1, but with no peripheral targeting (Figure S2A). We next expressed each of the two identifiable domains in the carboxy-terminus of OCRL1 (ASH and Rho-GAP domains [10], [11]), however GFP-tagged chimeras with each domain showed no junctional targeting (Figure S2B). Thus, the junctional localization of OCRL1 requires the presence of both ASH and Rho-GAP domains, neither of which are sufficient on their own for junctional localization. This requirement is the same as that for the interactions of OCRL1 with APPL1 [23] and IPIP27A/B (also called Ses1/2) [20], [21]. Overall, these results confirm that OCRL1 targets intercellular junctions.

### OCRL1 interacts with adherens and tight junction proteins in polarized epithelial cells

**Figure 4:**
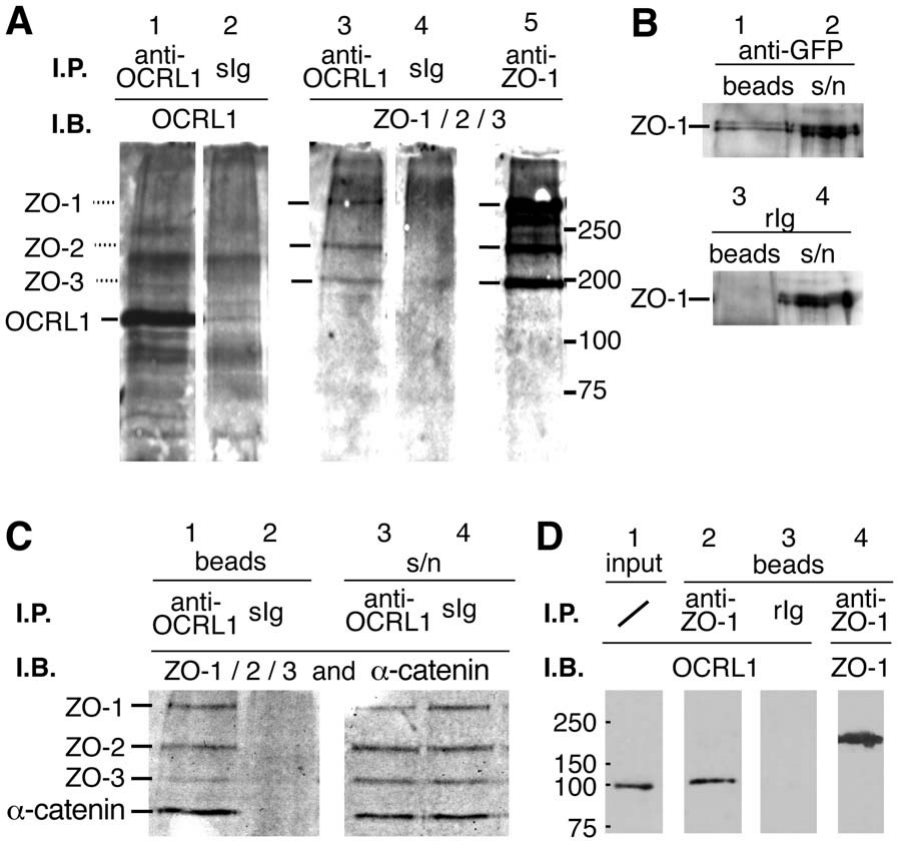
OCRL1 interacts with ZO-1, ZO-2, ZO-3 and α-catenin in polarized epithelial cells. (**A**) OCRL1 forms complexes with junctional proteins in MDCK cells**.** OCRL1 antibodies (lanes 1 and 3) were used for immunoprecipitation in parallel with respective controls – sheep immunoglobulin (sIg, lanes 2 and 4) and anti-ZO-1 (lane 5) from pre-cleared MDCK cell lysates. Precipitated proteins were run on SDS-PAGE gels, and probed with antibodies to OCRL1 (lanes 1 and 2) or with a mixture of non-cross-reacting antibodies to ZO-1, ZO-2 and ZO-3 (lanes 3, 4 and 5). Molecular weight markers are indicated from 75 to 200 kD. (**B**) GFP-tagged OCRL1 forms complexes with a significant proportion of ZO-1. GFP was precipitated from Caco-2 cells that had been transfected with GFP-OCRL1, and immunoblotted for ZO-1, as in A. Rabbit immunoglobulin (rIg) was used as control. (**C**) OCRL1 forms complexes with both tight and adherens junction proteins in MDCK cells. Antibodies to OCRL1 (lanes 1 & 3) were used for immunoprecipitation in parallel with control sheep Ig (lanes 2 & 4) from pre-cleared MDCK cell lysates. Precipitated proteins (lanes 1 & 2) and supernatants (lanes 3 & 4) were run on SDS-PAGE gels and probed with a mixture of non-cross-reacting antibodies to ZO-1, ZO-2, ZO-3 and α-catenin. In this experiment, specific precipitation of OCRL1 was highly efficient, with almost complete deletion of detectable OCRL1 from the supernatant (data not shown). (**D**) ZO-1 complexes in MDCK cells contain OCRL1. Antibodies to ZO-1 (lanes 2 and 4) were used for immunoprecipitation in parallel with control rabbit Ig (lane 3) from pre-cleared MDCK cell lysates (lane 1). Precipitated proteins (lanes 2 to 4) and input (lane 1, equivalent to 5% of what was added to the beads) were run on SDS-PAGE gels and probed with antibody to OCRL1 (lanes 1 to 3) or to ZO-1 (lane 4). In this experiment, α-catenin was also readily detected in anti-ZO-1 complexes (data not shown).

Given the co-localization of OCRL1 with both ZO-1 and α-catenin at early times after plating of epithelial cells, we looked for the presence of complexes between them. We first immunoprecipitated OCRL1 from MDCK cells grown for 24 hours (Figure 4A), and examined complexes for zonula-occludens proteins. Compared to control immunoglobulin (lane 2), antibodies to OCRL1 precipitated one major OCRL1 band of the expected molecular weight (lane 1). This immunoprecipitate was enriched for ZO-1, ZO-2 and ZO-3 (lanes 3 and 4). ZO-2 and ZO-3 are known to form complexes with ZO-1 [27], a finding that we replicated in a separate immunoprecipitation with antibodies to ZO-1 (lane 5). This observation was repeated using Caco-2 cells (Figure S3). We next transiently transfected Caco-2 cells with GFP-OCRL1, which partly co-localizes with ZO-1 (Figure 3A). This GFP-OCRL1 formed complexes enriched in ZO-1 (Figure 4B, lanes 1/2) compared to control immunoglobulin (lanes 3/4).

We next asked if OCRL1 complexes contain α-catenin in addition to ZO-1/2/3. Repeating a precipitation with antibodies to OCRL1, we found that OCRL1-positive complexes also contained α-catenin (lane 1 compared to lane 2) in addition to ZO-1/2/3. This complex does not represent precipitation of all junctional components, but rather a subset, since when the same complexes when run out on additional blots and probed with individual antibodies to cortactin, E-cadherin, β-catenin and p120-catenin, all were shown to be absent, while supernatants were strongly positive for each antigen (data not shown). To further confirm the interaction between OCRL1 and ZO-1, we carried out reverse precipitations with anti-ZO-1 (Figure 4D). These complexes contained OCRL1 (lane 2), which was not seen in control precipitations (lane 3), and these complexes also contained α-catenin (data not shown), consistent with previous reports that ZO-1 and α-catenin are both components at primordial junctions [2]. Parallel precipitations of OCRL1 from cells grown to higher confluency failed to include ZO-1 in complexes (data not shown), indicating that the co-precipitation of ZO-1 and OCRL1 correlates with junctional targeting of OCRL1 in that both are restricted to cells at the early stages of confluent growth. In summary, we have shown that OCRL1 is present in complexes with a subset of junctional proteins (ZO-1/2/3 and α-catenin), but it does not interact with other junctional proteins (cortactin, E-cadherin, β-catenin and p120-catenin).

### OCRL1 is important for normal development of polarized epithelial monolayers

**Figure 5:**
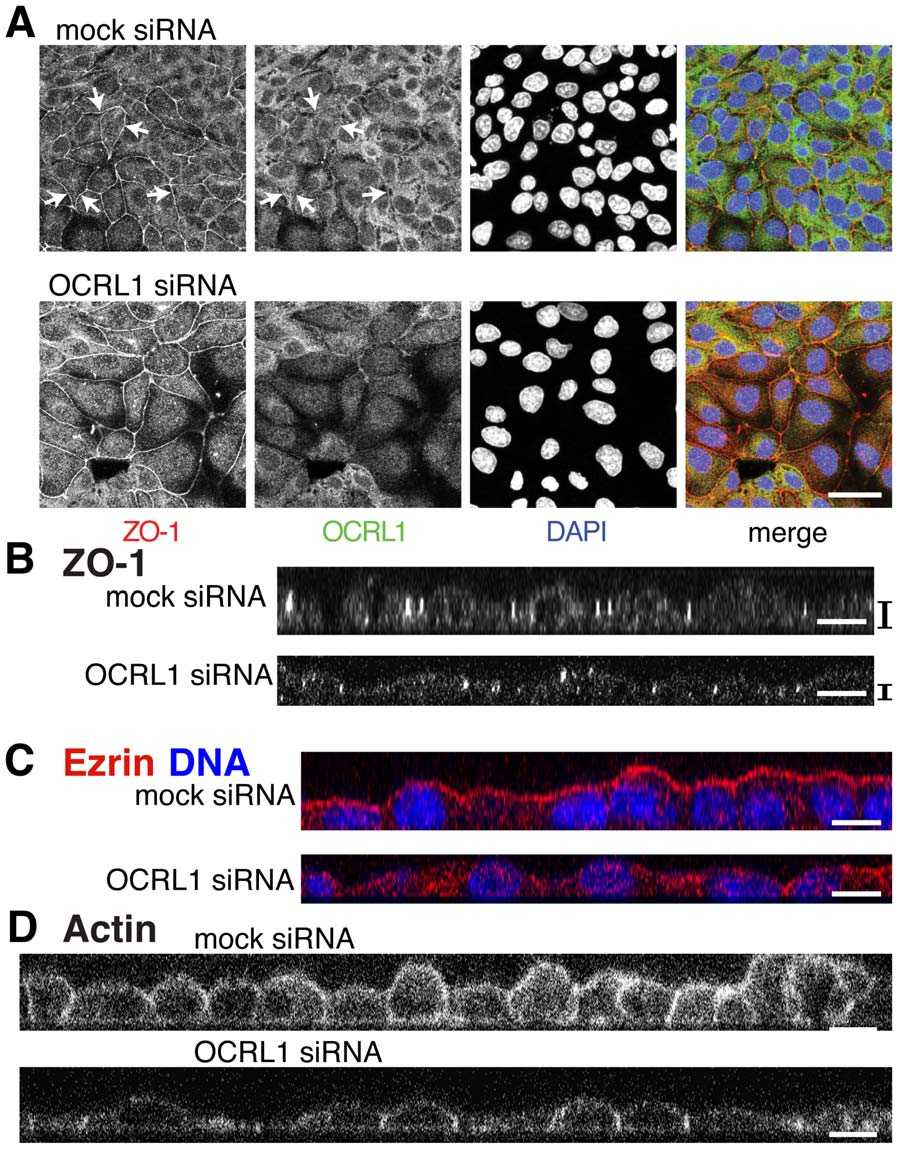
OCRL1 depletion inhibits maturation of polarized epithelial monolayers. (**A**) MDCK cells were treated over 72 hours to silence OCRL1 expression with a single siRNA duplex or with a control siRNA duplex, as described in Materials and Methods, fixed and immunostained as in Figure 1. ZO-1 staining delineates cell borders, which shows gaps because this is a single confocal section. OCRL1 depletion was associated with an increase in the area of cells (as seen from their borders and the spacing of nuclei) that was typical of the x1.67±0.11 (n = 4) increase seen with four different OCRL1-specific duplexes. Junctional OCRL1 was only detected faintly (arrows), which is consistent with confluency >70% (see Figure 1F). (**B**) XZ sections of ZO-1-stained MDCK cells, as in A. Mean height above substrate of total cellular ZO-1 staining was: 5.1 µm for controls and 2.9 µm after depletion of OCRL1 (indicated on right), a ratio of 1.74∶1. (**C**) XZ sections of cells treated over 96 hours to silence OCRL1 expression and with control siRNA duplexes, stained for ezrin (red) and DAPI (blue). (**D**) XZ sections of cells treated over 96 hours to silence OCRL1 expression and with control siRNA duplexes, stained with fluorescently labeled phalloidin to demonstrate F-actin. All scale bars 10 µm.

To test whether OCRL1 has a role in epithelial maturation, we depleted OCRL1 by RNA silencing in MDCK cells. In various cell types, we achieved ≥85% depletion of OCRL1 in comparison to cells treated with an irrelevant RNA duplex, as assessed by Western blots (Figure S4A). Because OCRL1 only targets junctions in cells that have only recently attained confluence, we depleted OCRL1 in cells growing at very low confluency (20%), as this was most likely to deplete OCRL1 before junctions start to form. We then allowed cells to grow for up to 3 days further. It is important to note that by the end of this growth period, OCRL1 could no longer be detected at junctions (Figure 1F). MDCK cells lacking OCRL1 occupied a larger surface area (Figure 5A). To demonstrate that the effect was specific, four different oligonucleotides specific for OCRL1 were tested individually in MDCK cells. All four achieved similar results, with an increase in cell area of x1.67±0.11 (n = 4). To accompany the shape change, there were fewer cells; cells on the dish counted by DAPI staining of nuclei precisely matched this increase in area (x 0.60±0.5). These results indicate that effects on cell shape/number were not an off-target effect of RNA interference. Assessment of the loss of cell height was made from XZ sections, which showed that height was reduced to an extent similar to the increase in cell area (Figure 5B), so that cell volume was unaltered. Counting cell number, we found that knock down of OCRL1 reduced proliferation of polarizing MDCK cells, an effect that persisted even if cells were diluted back to low confluence (Figure S4B). Thus, lack of OCRL1 produced fewer MDCK cells that were flatter and occupied a larger surface area.

The effect on cell shape was also seen in two other polarized epithelial cell lines: Caco-2 cells (Figure S4C and S4D) and human corneal epithelial cells (Figure S4E) [28], [29]. We tested to see if the reduced cell number might be caused by increased apoptosis, and found apoptotic bodies associated with knock-down of OCRL1 at 72 hr were 0.03%±0.006 of total MDCK cells, compared to 0.02%±0.001 in controls. This difference is too small to explain the reduced cell numbers. Compared to polarized epithelial cells, HeLa cells lacking OCRL1 did not change cell number, or their cross-sectional area, as indicated by the spacing of nuclei in control and knock-down cells (Figure S4F), and cell proliferation in HeLa was unaffected, which is consistent with other reports on non-polarized mammalian cells [30].

Given that OCRL1 is detected at nascent junctions, we examined whether OCRL1 was important for the initial phase of junctional development. First, in the images of ZO-1 already shown above (Figures 5A & S4C/D/E), we found that lack of OCRL1 did not reduce strong linear staining of cellular junctions by ZO-1. Similarly, the adherens junction component E-cadherin did not redistribute from junctions to internal pools, although cells lacking OCRL1 are more densely stained, presumably because of their reduced cross-sectional area (Figure S5A). Even though the distributions of markers of adherens and tight junctions appeared normal, we used calcium switch assays to determine if lack of OCRL1 affects the function of newly forming junctions [31]. Lack of OCRL1 had no effect on the rise in trans-epithelial resistance over 24 hours after re-addition of calcium (data not shown), suggesting that lack of OCRL1 does not affect the initial phase of junction formation for epithelial cells growing as sheets.

In addition to components of the junctions themselves, we also determined if OCRL1 has a role in the distribution of polarized membrane markers. The marker ezrin polarizes to the apical domain during epithelial maturation [32], and was highly enriched at the apical surface of control cells; however, ezrin was only rarely and weakly found at the apical surface of cells lacking OCRL1 (Figure 5C). The apical marker gp135/podocalyxin [33] also showed a loss of polarization, accompanied by reduced expression (Figure S5B). Along with the lack of apical ezrin and gp135, the normal apical enrichment of F-actin (for example [34]) was inhibited (Figure 5D). After 96 hours in culture, control cells showed prominent enrichment of F-actin in the apical compartment, with bright linear staining along the slightly domed apical surface (Figure 5D, top). In contrast, in cells lacking OCRL1 actin did not redistribute to the apical compartment, and instead the highest concentration of F-actin was at the cortical ring coinciding with intercellular junctions (Figure 5D, bottom). These results indicate that OCRL1 has a role in the maturation of polarizing epithelial cells, but not in the initial formation of junctions.

### Recovery of epithelial development upon OCRL1 re-expression is dependent on 5′-phosphatase activity

**Figure 6:**
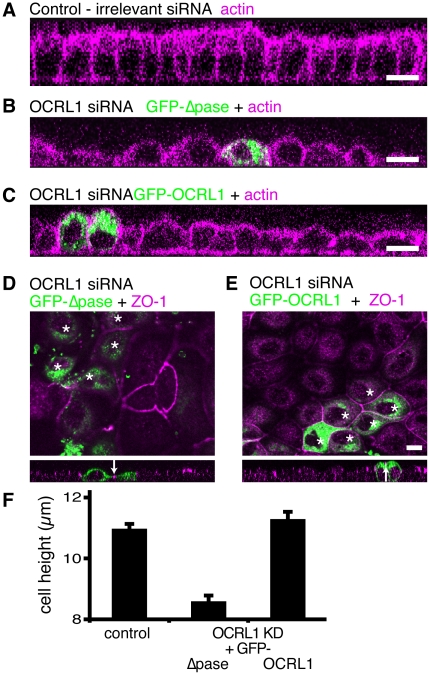
Increase in cell and junctional height with OCRL1 re-expression is dependent on 5′-phosphatase activity. MDCK cells were treated over a period of 96 hours (**A**) with control, non-targeting siRNA, (**B–E**) with four pooled OCRL1 siRNA duplexes. At 72 hours, cells were transfected either (**B, D**) with GFP-OCRL1-Δphosphatase (“GFP-Δpase”) , or (**C, E**) with GFP-OCRL1, and grown for a further 24 hours. (**A–C**) XZ sections counterstained for F-actin (cyan). (**D, E**) the highest confocal section where ZO-1 (cyan) appeared. Note that these images are single confocal sections, so lack of ZO-1 indicates that cells are flat and their ZO-1 is below the plane of section. Asterisks indicate transfected cells adjacent to one another. XZ sections are shown below, each including an adjacent pair of transfected cells and the junction between them (arrows). (**F**) Images of cells from (A–C) were used to determine total cell height of 50 transfected cells. Results are from a representative of 3 experiments ± s.e.m. Cells re-expressing Δpase were less tall than other groups (p<10^−12^). Scale bars are 10 µm.

We next looked for possible effect of cell shape of expressing GFP-tagged OCRL1. In untreated cells with endogenous OCRL1, over-expression of GFP-OCRL1 compared to GFP alone had a marginal impact on cell height (11.8 µm, s.e.m. 0.16 *cf* 10.7 µm, s.e.m. 0.31, p = 0.002, n = 50). We then re-expressed GFP-OCRL1 in cells where its expression was silenced. MDCK cells (canine in origin) with silenced OCRL1 were transfected with human OCRL1 constructs that are resistant to canine-specific siRNA oligonucleotides (Figure 6A/B/C). OCRL1 lacking the 5′-phosphatase domain localized to the Golgi and cytoplasmic puncta as described previously [12], but caused no gain of height (Figure 6B). In these cells the height of junctions above the substrate was also unaffected (Figure 6D). By comparison, cells re-expressing full-length OCRL1 had increased height (Figure 6C and 6E), almost identical to untreated cells (Figure 6F). Furthermore, where adjacent cells both express GFP-OCRL1 (asterisks and arrow in Figure 6E), not only were the cells taller, but the junctions as identified by ZO-1 were in a plane several microns higher than in untransfected neighbours. This indicates that the phosphatase activity of OCRL1, likely through modulation of PIPs, contributes to the increase in height of MDCK cells from flat/cuboidal to tall/columnar.

### OCRL1 depletion causes disruption of 3D MDCK cyst formation

**Figure 7:**
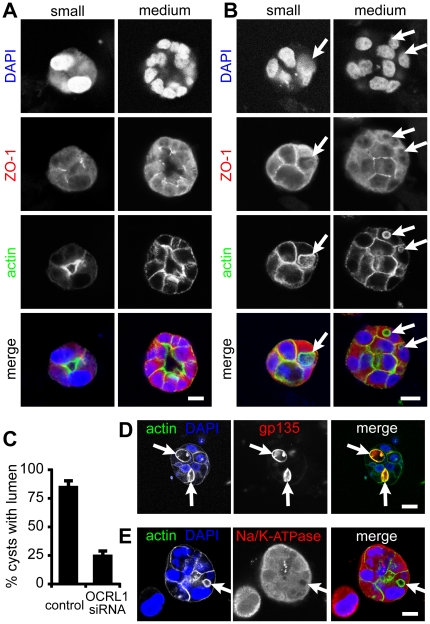
OCRL1 depletion interferes with lumen formation in MDCK cyst morphogenesis. (**A**) MDCK cells were treated twice with control, non-targeting siRNA for 72 hours prior to being seeded into collagen/matrigel and allowed to proliferate for 4 days. Cells were fixed with 3% paraformaldehyde, permeabilized, blocked and processed for staining with DAPI, anti-ZO-1 and phalloidin (blue, red and green in merge respectively). Images (single confocal sections) are from either small clumps (2 to 4 nuclei in widest cross-section), or medium-sized clumps (approx. 10–15 nuclei in widest cross-section). Size bars  = 10 µm. (**B**) As in A, but with four duplexes targeting OCRL1. Arrows indicate actin-rich spherical, intracellular vacuoles and the positions these occupy in alternately stained images. (**C**) Phalloidin images from two independent experiments were analysed for lumen formation. A total of 84 cysts were counted. 85% of control siRNA treated cell clumps were hollow cysts with lumens. In comparison, lumen formation was only seen in 25% of clumps of cell with silenced OCRL1. Bars show range from 2 experiments; t-test of these data showed the difference was significant (p<0.01). (**D and E**) MDCK cells were treated as in B. and stained with DAPI (blue) and phalloidin (left-hand panels, green in the merge), and antibodies either to gp135 (podocalyxin) or to sodium/potassium ATPase (right-hand panels, red in the merge). Scale bars are 10 µm.

As a further test for the function of OCRL1 in polarized epithelial cells, we examined its role in cyst formation in a three-dimensional tissue culture model, partly because cell growth in 3D is more sensitive for showing phenotypes associated with abnormal junctions than growth of cells in 2D [35]. MDCK cells were treated with control irrelevant siRNA or OCRL1-specific siRNA as before, and then seeded in collagen/matrigel gels, in which they developed into cysts. After 4 days growth, control cells formed cysts with single, large lumens (Figure 7A). In contrast, MDCK cells lacking OCRL1 typically remained as solid clumps, failing to form lumens (Figure 7B). Only 25% of OCRL1-depleted cysts formed lumens, compared to 85% in control experiments (Figure 7C). The reduction in lumen formation in cells depleted of OCRL1 was not the result of reduced growth, as there were large clumps of these cells, and even these showed greatly reduced lumen formation. Apart from the changes in lumen formation, there were intracellular changes for both actin and ZO-1. In the smallest cysts (<10 cells), under control conditions actin was enriched just beneath apical membranes surrounding the lumen (Figure 7A). As these cysts developed further (>20 cells), actin was also found in the cortex laterally, and to a much lesser extent basally (Figure 7A). In contrast, after depletion of OCRL1, actin was enriched laterally and basally even in the smallest cell clumps (Figure 7B). In addition, some cells contained spherical vacuoles heavily lined by actin (Figure 7B). Such structures were never seen in control cells, and were positive for gp135/podocalyxin, but negative for the basolateral marker sodium/potassium ATPase (Figures 7D and E). Although these vacuoles came close to the plasma membrane, they showed no direct continuities with it. With regard to ZO-1, depletion of OCRL1 led to this junctional marker being localized away from apical regions into broader regions of cell-cell contact (Figure 7B). These results show that when grown under more physiological conditions in matrigel, epithelial cells lacking OCRL1 have an extensive defect in cell morphogenesis, including effects on ZO-1 that were not seen when the same cells were grown in sheets on plastic.

## Discussion

Our key finding is that loss of OCRL1 inhibits epithelial maturation in 2D and 3D cultures. The effects are striking and reproducible in multiple epithelial cell types. The requirement for the 5′-phosphatase domain for rescue of cell height (Figure 6) suggests that removal of phosphoinositides by OCRL1 may be critical in the process by which epithelial cells develop. There are few previous studies of OCRL1 in epithelial cells, and these have not found the protein at junctions [25], [36]. The targeting of GFP-tagged constructs to the lateral compartment of both Caco-2 and MDCK cells only in the most apical sections, and not the entire lateral compartment (Figure 3), supports our conclusion that OCRL1 targets an apical junctional complex. This differs from what we were able to detect with endogenous OCRL1. In cells that had been recently plated, at which time the cells were still quite flat, OCRL1, together with α-catenin, was present laterally in all confocal sections, differing from ZO-1 which was apical. However, in cells plated for longer periods, where apical junctional complexes have matured and segregated, we did not detect lateral OCRL1 (compare Figures 1C and 1F). This might be the reason why lateral targeting of OCRL1 has been missed by others. It is possible that, like other junctional antigens, OCRL1 is not detected because it is fixed into complexes that block access to antibodies. However, mild detergent extraction prior to fixation did not increase antigen exposure (data not shown) [37]. Although, the lack of OCRL1 at junctions in cells grown to higher density (Figure 1F and Figure 5) remains unexplained, it correlates with our data on co-precipitation of ZO-1 with OCRL1, in that ZO-1 was absent from OCRL1 complexes when cells were grown to higher confluency than in Figure 4 (data not shown).

Junctional targeting by OCRL1 is admittedly weak, but this pattern is still functionally relevant, as it resembles similar targeting by other proteins that act at junctions [29], [38]. OCRL1 has many binding partners [11], [16], [17], so only a small pool may target junctions. Junctional targeting by OCRL1 required both ASH and RhoGAP domains in tandem, the same requirement as for the interaction of OCRL1 with APPL1 and the recently described endocytic proteins IPIP27A/B (also called Ses1/2), which all share an OCRL1-interacting “F&H” motif [20], [21]. It is possible that the interaction partner of OCRL1 at junctions contains a F&H-like sequence [39].

The relevance of the small junctional pool of OCRL1, and of its interactions with junctional proteins is not clear. While it is possible that the functionally important pool of OCRL1 is targeted to junctions and binds to junctional proteins, this remains to be proven, and would need a detailed molecular dissection of the interactions of OCRL1 with junctional proteins and its ability to rescue phenotypes. Alternative explanations include that the interactions of OCRL1 with ZO-1/2/3 and α-catenin do not mediate targeting to junctions. For example, if OCRL1 exerts its primary effect via the cytoskeleton, it might be interacting with the pools of ZO-1/2/3 and α-catenin that are associated with the cytoskeleton, which would explain why we did not find other components of the cadherin-catenin complex in OCRL1 complexes. Additionally, if OCRL1 acts via membrane traffic, the critical pool of OCRl1 may be on post-Golgi carriers [16], or on recycling endosomes carrying junctional proteins [40].

There are many different possible mechanisms by which OCRL1 might be required for normal development of polarized epithelial cells, affecting both cell shape and proliferation. We do not find any excess of binucleate cells (data not shown) which indicates that a failure of cytokinesis is not the likely mechanism [30], [41]. OCRL1 may directly affect one of the junctional proliferative signalling pathways to account for the reduced cell number. The lack of this effect in non-polarized cells (Figure S4F), indicates that any signal would most likely be downstream of a pathway specific to apical junction complexes [1]. Alternately, the effect on cell number (Figure 5) could be indirect, acting via several pathways that also act on cell shape (actin polymerization, membrane traffic, Cdc42, or direct interactions of PIPs), which we discuss below.

Actin polymerization is important for junctional maturation [42], and is regulated by OCRL1 [13], [14], [15]. In 2D and 3D cultures, lack of OCRL1 altered actin distribution (Figures 5 and 7). Our finding that OCRL1 is in complexes with α-catenin, which affects actin polymerization [42], supports the idea that actin polymerization near junctions could be a target of OCRL1. Also, the shape change with lack of OCRL1 is similar to lack of Tmod3 [34], a tropomodulin acting with actin to mediate epithelial maturation [43].

Another mechanism by which OCRL1 might be involved in development of polarized epithelial cells is by regulating membrane traffic of junctional components, similar to the known effects of OCRL1 on sorting of lysosomal enzymes [25], [44], [45]. Our constructs expressed the OCRL1 b isoform, which has multiple interactions with the clathrin endocytic machinery [22], [23], [24], which is involved in recycling (hence remodelling) of junctional components [40], as well as interactions with many rabs [16], including the junctional Rab8 [40]. Junctional OCRL1 might also affect traffic via the exocyst, which localises to junctions and is activated by PI45P_2_ [46], [47]. A specific indication that OCRL1 affects traffic is that depletion in 3D cultures inhibits lumen formation in cysts, with the appearance of large intracellular vacuoles positive for apical markers. These may derive from vacuolar apical compartments (VACs) [48], [49], which are normal intermediates in the Cdc42-dependent apical exocytic pathway that forms the lumen by hollowing out a central space between 2 or more cells growing in matrigel [50].

The knock-down phenotype in 3D cultures is similar to that of cysts lacking Cdc42, which may act via wrong positioning of apical membrane domains [51]. This similarity might be explained in part through loss of the OCRL1-Cdc42 interaction [11], [17]. Large vacuoles positive for apical markers are not only seen with loss of Cdc42, but also with loss of PTEN, Annexin-2, and atypical protein kinase C [4]. All these proteins together are required to segregate PI45P_2_ on the apical domain from PI345P_3_ basolaterally, a process that is required for apical delivery of VACs. OCRL1 might therefore be involved in segregating PI45P_2_ from PI345P_3_, which is also a target of its 5-phosphatase activity [52]. Interestingly, in Sertoli cells of Inpp5b knockout mouse, enlarged actin-lined vacuoles are also observed, which suggests similar phenotypes exist for the loss of OCRL1 and Inpp5b [53].

A more direct role for PI45P_2_ at junctions may arise from its interaction with a sub-group of PDZ domains, in particular the first two PDZ domains of ZO-1, which are predicted to produce significant binding to the lipid in vivo [3]. During maturation of junctions, OCRL1 might dephosphorylate junctional PI45P_2_, made by PI4P 5-kinase-β that binds to E-cadherin [5], as a step necessary for the release of ZO-1 from primordial junctions. Supporting the idea that PI45P_2_ has a key role in junction maturation, inhibition of phosphatidylinositol 3-kinase in epithelial cells (which presumably elevates PI45P_2_), induces a similar morphological effect to knock-down of OCRL1 [54]. Given the large number of possible pathways downstream of OCRL1, further work with both 2D and 3D cultures will be required to dissect how OCRL1 acts in development of polarized epithelial cells.

How do these findings relate to Lowe Syndrome? Intercellular junctions are essential for the function of the cell types affected by loss of OCRL1. Glia myelinating small neurons form tight junctions required for normal impulse conduction [55]. Renal tubular disorder can result from junction dysregulation [56]. Lens fibre cells, the most sensitive cell type to loss of OCRL1, form extensive junctions highly enriched with α- and β-catenins [57], and their architecture requires tropomodulins [58]. Our future work will focus on the mechanism by which OCRL1 acts at junctions, to identify proteins that might be inhibited or activated to compensate for loss of OCRL1 in Lowe Syndrome.

## Materials and Methods

### Materials and antibodies

Affinity purified sheep antibodies to the amino-terminal 240 amino acids of OCRL1 are as previously described [12]. Antibody production was by PTU/BS (Penicuik, UK), with approval of the local research ethics committee for the Moredun Research Institute and reviewed by the local research ethics committee for PTU/BS (approval number 275), all under Home Office approval (project license #30-3464). Other antibodies described previously are: anti-ZO-1/2/3 [59], and anti-podoclyxin/gp135 [33]. Antibodies to α-catenin and all other materials, unless otherwise stated, were obtained from Sigma-Aldrich.

### Molecular biology

GFP tagged human OCRL1 (wild-type, isoform b = 893 amino acids), GFP-OCRL1Δphosphatase lacking the phosphatase domain (Δ237–539), and GFP-Inpp5B are as described [9], [12]. OCRL1 amino-terminus consisted of amino acids 1-501; OCRL1 carboxy-terminus consisted of amino acids 501 to 893. These constructs were created by excising coding sequence downstream or upstream of the endogenous EcoRI site coding for R500 and I501 in OCRL1. For the ASH and RhoGAP domains alone, residues 547**–**723 and 724**–**893 respectively were cloned downstream of GFP.

### Mammalian cell culture and transfection

MDCK cells [37] and Caco-2 cells [60] were grown at 37°C and 5% CO2 in DMEM containing 10% or 20% fetal calf serum respectively, 100 µg/ml streptomycin and 100 µg/ml penicillin (regular medium). For visualization of GFP-tagged constructs, cells at approximately 20**–**30% confluence were transfected with 0.4 µg plasmid DNA in 35 mm glass bottom dishes (MatTek Corporation) with 10 µl Lipofectamine-2000 (Invitrogen). DNA/lipofectamine complexes were made in serum-free conditions and incubated with cells in 2 ml regular medium. After 6 hours of incubation, cells were washed with PBS and replaced in regular medium overnight. GFP-tagged constructs were analysed 16**–**24 hours after transfection.

For seeding into matrigels, MDCK cells treated with siRNA were trypsinized, diluted 1∶2 with serum-containing medium to inactivate trypsin, spun, and then resuspended to a single cell suspension and counted. 260 µl collagen-Matrigel master mix was prepared by neutralizing 152.5 µl of ice-cold solution containing 1 mg/ml calf skin type I collagen (Sigma; C8919) with 25 µl 10x Dulbecco's modified Eagle's medium, 5 µl HEPES 1 M (pH 7.4), and 6.25 µl of 2 M NaOH that was then mixed with 25 µl of 100% fetal bovine serum and 41.25 µl of Matrigel (growth factor reduced; BD Biosciences). 100 µl collagen-Matrigel mix was plated in a well of a 48-well dish containing a coverslip for 1 h at 37°C during which time it solidified into a gel. Approximately 30,000 cells were seeded into the remaining 160 µl of collagen-Matrigel, allowed to solidify for 3 hours and then covered with low glucose tissue culture medium. Cysts were allowed to develop over 3**–**4 days, with replacement of medium every 2 days. For fluorescence labelling, cells were fixed with 3% paraformaldehyde for 20 minutes at room temperature and were then washed twice with PBS. Cells were blocked and permeabilized for 30 minutes at room temperature with 2% BSA, 1% Triton X-100, and 0.1% SDS in PBS, followed by incubation overnight at 4°C with antibodies (rabbit anti-ZO-1, mouse anti-gp135 or sodium/potassium ATPase). After three washes with 2% BSA, 1% Triton, 0.1% SDS in PBS, the samples were incubated with secondary antibodies, cy5-phalloidin and DAPI. After three washes with PBS, cells were mounted with Mowiol. For quantification of the different structures in clumps/cysts described in Results, at least 20 low-magnification images of cells stained with phalloidin were used for each cell line/condition.

### Immunoprecipitation

Cells grown in 35 mm tissue culture wells were washed three times in PBS. Cells were then placed in ice-cold 500 µl lysis buffer (10 mM HEPES, 142.5 mM KCl, 0.2% NP-40, 2.5 mM sodium pyrophosphate, 1 mM β-glycerol phosphate, 1 mM sodium orthovanadate, 100 µM CaCl_2_, pH 7.4) and scraped off the bottom of the wells. All lysis steps and subsequent washes were performed in the presence of protease inhibitor cocktail (Sigma) and on ice. Lysates were then incubated with pre-clearing Protein G beads in lysis buffer for 30 minutes at 4°C. Lysates were then added to washed Protein G beads pre-coated overnight with stated antibodies. Lysate and bead mixes were then placed on a rotator, incubated at 4°C for two hours, and then centrifuged at 7,500 x g and the supernatants were saved. Beads used for the immunoprecipitation were then washed and centrifuged at 7,500 g six times. After washing steps, sample buffer was added to the beads. 2x sample buffer was also added to supernatants (1∶1). Beads and supernatants were boiled for ten minutes and stored at −20°C. Loading of these samples was always 1/3 of the beads and 1/100 of the supernatant.

### SDS-PAGE and Western blotting

Samples were run on 10% polyacrylamide minigels for 90 minutes at 150 V at 4°C. Proteins were transferred by wet blotting for 45 minutes at 300 A to PVDF membranes, blocked in 1x PBS in 0.05% Tween20 supplemented with 5% powdered milk (Marvel) and incubated with stated primary antibodies. For ZO-1 Western blots, 7.5% polyacrylamide SDS-PAGE gels were transferred to nitrocellulose through wet blotting for 3 hours at 350 A at 4°C. Nitrocellulose membranes were then treated with Amidon Black solution for 5 minutes and destained in 20% methanol/7.5% acetic acid. Primary antibody incubations were for one hour at room temperature or overnight at 4°C. PVDF and nitrocellulose membranes were then washed with PBS/Tween20 three times before a one hour incubation at room temperature with relevant HRP-conjugated secondary antibodies (Dako). After 6 washes with PBS/Tween20, PVDF membranes were visualized by Enhanced Chemiluminescence (Amersham Biosciences) using Fuji medical x-ray film.

### Immunofluorescence microscopy

Cells were first washed with PBS and subsequently either fixed with 4% paraformaldehyde on ice for 20 minutes or with methanol for 5 minutes at −20°C, as stated. After fixation with methanol, cells were washed three times with PBS and then left in PBS for 15 minutes for rehydration. Cells fixed by paraformaldehyde were then permeabilized by 0.2% Triton-X100 in PBS. To attempt to enhance exposure of epitopes in junctional complexes (see Figure 1), cells were pre-exposed to detergent (0.1% TX-100) on ice for 2 minutes prior to fixation [37], but this did not alter the staining pattern for OCRL1, and so was not routinely performed. Blocking of the cells was carried out in PBS in the presence of 1 mg/ml bovine serum albumin and 0.2% TX-100 for 1 hr. Primary antibodies were incubated with the cells in PBS in the presence of 0.2% Triton X-100 overnight and washed three times. Secondary antibodies (Invitrogen, unless stated) were then incubated in PBS plus 0.2% TX-100 and cells were washed three times, before one wash with water and mounting in Vectashield (Vector Laboratories, Inc) for imaging on a confocal microscopy system (AOBS SP2; Leica) at room temperature (63x NA 1.4 objective) using LCS software (Leica) for acquisition.

### RNA interference

Cells were transfected according to manufacturer's instructions. Briefly, non-targeting, Allstars negative control siRNA (Qiagen #1027281), or pooled human OCRL1 siRNA sequences #1: CUUUCCGGUACCUUCGUUCUU, #2: AAAGCCUUAGUCUUCUUCGUU, #3: UUUGAUGAGACCCUCCCGCUU, #4: UAUCGACACUGAUCCUUUCUU (SmartPool, Dharmacon) were made into complexes with 5 µl Oligofectamine (Invitrogen) in serum-free medium. In the case of RNA silencing in MDCK cells, the canine equivalents of the same oligonucleotides were used together or individually. In all cases, final total siRNA duplex concentration was 100 nM. After initial plating at 10% confluence in 2 ml regular medium (without penicillin/streptomycin) on 35 mm glass bottom dishes (MatTek Corporation), cells were treated twice (at 24 hours and again 24 hours later), with lipofectamine/siRNA complexes, and then analysed 48**–**72 hours after first siRNA treatment.

### Transepithelial resistance

Trypsinized cells (48 hours after commencement of control or OCRL1 siRNA) were plated in excess, onto an 8-well Electric Cell-substrate Impedance Sensing (ECIS, Sislab) 8-well slide in low-calcium medium (Spinner modification of MEM, Sigma-Aldrich). After cells had become adherent to the bottom of the wells, excess cells were removed through careful aspiration and cells were grown in fresh low calcium medium overnight at 37°C. Medium was replaced with regular DMEM, and after 15 minutes, measurement of the impedance was commenced for up to 24 hours. These results were used to indicate trans-epithelial resistance of the monolayer.

## Supporting Information

Figure S1 **Antibody staining of OCRL1 at junctions is lost with OCRL1 depletion by RNA interference.** MDCK cells were treated for 48 hours to silence OCRL1 expression and with control RNA duplexes (as described in Materials and Methods, also see Fig S4 below) were immunostained for OCRL1 and examined by confocal microscopy. While controls show OCRL1 at junctions and internally in the region of the Golgi apparatus, after knock-down both types of staining are lost, indicating junctional staining is specific for OCRL1 protein. Scale bars are 10 µm.
(TIF)

Figure S2 **The ASH domain or the Rho-GAP domain of OCRL1 alone does not mediate junctional localization.** (**A**) GFP-OCRL1–amino-terminus and GFP-OCRL1–carboxy terminus expressed in HeLa cells, as in Figure 3. While neither targets the periphery, the carboxy-terminus targets internal membranes as seen for polarized cells (see Figure 3E). (**B**) GFP-tagged ASH and Rho-GAP domains were expressed in Caco-2 cells, as in Figure 3. Neither construct targets junctional regions, as identified by ZO-1 staining. Scale bars are 20 µm.
(TIF)

Figure S3 **Endogenous ZO-1 immunoprecipitates with OCRL1 in Caco-2 cells.** Caco-2 cell lysates were prepared as in Figure 4A. OCRL1 (lanes 1 & 2) and irrelevant antibodies (normal sheep immunoglobulin – sIg) (lanes 3 & 4) were used to precipitate proteins from a pre-cleared Caco-2 cell lysate. Precipitated proteins (30%, lanes 1 & 3) and unbound supernatants (1%, lanes 2 & 4) were separated by SDS-PAGE, and probed with antibodies to ZO-1, which runs as a doublet (arrow), only seen with anti-OCRL1.
(TIF)

Figure S4 **OCRL1 depletion by RNA interference in different cell lines.** (**A**) HeLa cells were treated over 72 hours to silence OCRL1 expression and with control RNA duplexes. Immunoblot shows levels of OCRL1 compared to loading controls (actin and β-tubulin for Caco-2 and HeLa respectively). Densitometry indicated reduction by ≥85%. Similar OCRL1 depletion was obtained in Caco-2, human corneal epithelial cells, and also in MDCK cells that had been treated with any one of 4 different siRNA duplexes tested (data not shown). (**B**) MDCK cells were plated in 24-well plates and then treated at 24 and 48 hours of growth to silence OCRL1 expression and with control siRNA duplexes, as described in Materials and Methods. Cells were removed by trypsinization and counted from 24 hours onwards. Inset: confluent cells removed from dishes at 96 hours were re-plated at 5% confluency for a further 24 hours. (**C**) Caco-2 cells were treated as in (B), fixed and immunostained as in Figure 1. Images are compressed confocal stacks. ZO-1 staining delineates cell borders. The increase in cross-sectional area for cells depleted of OCRL1 (bottom) compared to mock treated cells (top) was 2.4-fold (s.e.m.  = 0.4, p<10^−18^) in multiple fields of cells taken from two separate experiments. Note that these cells have been growing to >70% confluence over 96 hours, so the absence of junctional OCRL1 is similar to that seen in Figure 1F. Asterisks indicate 3 cells unaffected by OCRL1 siRNA. (**D**) XZ sections of ZO-1-stained cells from (B). The mean cell heights in these XZ sections (arrows at right-hand side) are: control  = 13.5 µm, OCRL1-slienced  = 5 µm, and over multiple fields the average height in cells depleted of OCRL1 = 55% (±7%, p<0.001) of control. Note these cells are taller than those in Figure 5B, mainly because they were growing at a greater confluency. (**E**) Human corneal epithelial cells were treated to silence OCRL1 expression, and immunostained for OCRL1 (as in B), which shows reduced expression except in a group of cells (asterisks). ZO-1 staining was used to delineate cell borders, showing a general increase in cross-sectional area in cells lacking OCRL1, compared both to the minority of cells where knock-down failed and to mock-treated controls. Note, only single XY sections are shown here, which explains why the ZO-1 staining is not continuous, unlike what is seen with a compressed stack (see Figure 5A) (**F**) HeLa cells were treated to silence OCRL1 expression, and immunostained for OCRL1 (as in B). Cell cross-sectional area (as determined from the spacing of nuclei stained with DAPI) is unaffected by loss of OCRL1. All scale bars 10 µm.
(TIF)

Figure S5 **Effect of OCRL1 knock-down on E-cadherin and on gp135.** (**A**) Caco-2 cells treated to silence OCRL1 (as in Figure S4C) were fixed and stained with anti-E-cadherin antibodies. As an internal control, cells in the lower right-hand corner (indicated by dashed line) resisted the knock-down of OCRL1, and had the same tall/columnar shape as wild-type cells (data not show). A similar pattern of E-cadherin at junctions and internal puncta is seen both in large, flat cells lacking OCRL1 and taller OCRL1+ve cells, although in the latter cells with far less cross-sectional area the puncta are crowded against the periphery. Scale bar 20 µm. (**B**) MDCK cells treated to silence OCRL1 were stained for the actin, DAPI and the apical marker gp135/podocalyxin. Experiment is similar to Figure 5A, but cells were fixed 24 hours earlier (*i.e.* 48 hours after initial siRNA treatment), explaining flatter shape. In control cells, actin and gp135 are enriched continuously across the apical domain,. In cells lacking OCRL1 there is failure of actin accumulation in the apical domain, and gp135 is either not expressed, or poorly expressed in just one segment of the apical domain, with some accumulation in a more basal region (arrow). Scale bars 10 µm.
(TIF)
