# Supplementary figures and images for: Lowe Syndrome Protein OCRL1 Supports Maturation of Polarized Epithelial Cells
Source: PLoS One. 2011 Aug 25;6(8):e24044. doi: 10.1371/journal.pone.0024044 (PMC3162020; doi:10.1371/journal.pone.0024044)

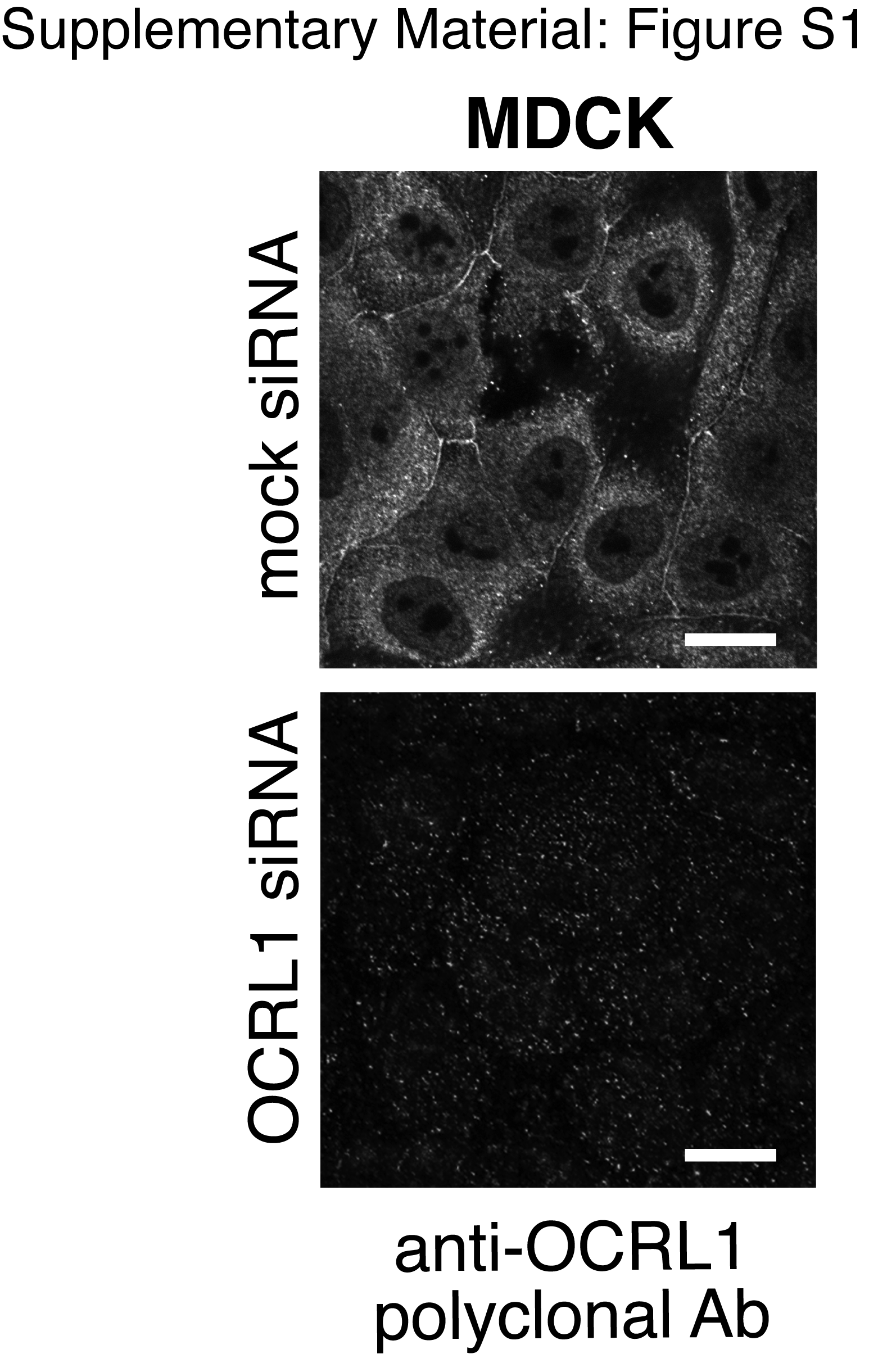

Supplement: Figure S1 — Antibody staining of OCRL1 at junctions is lost with OCRL1 depletion by RNA interference. MDCK cells were treated for 48 hours to silence OCRL1 expression and with control RNA duplexes (as described in Materials and Methods, also see Fig S4 below) were immunostained for OCRL1 and examined by confocal microscopy. While controls show OCRL1 at junctions and internally in the region of the Golgi apparatus, after knock-down both types of staining are lost, indicating junctional staining is specific for OCRL1 protein. Scale bars are 10 µm. (TIF) [file pone.0024044.s001.tif]

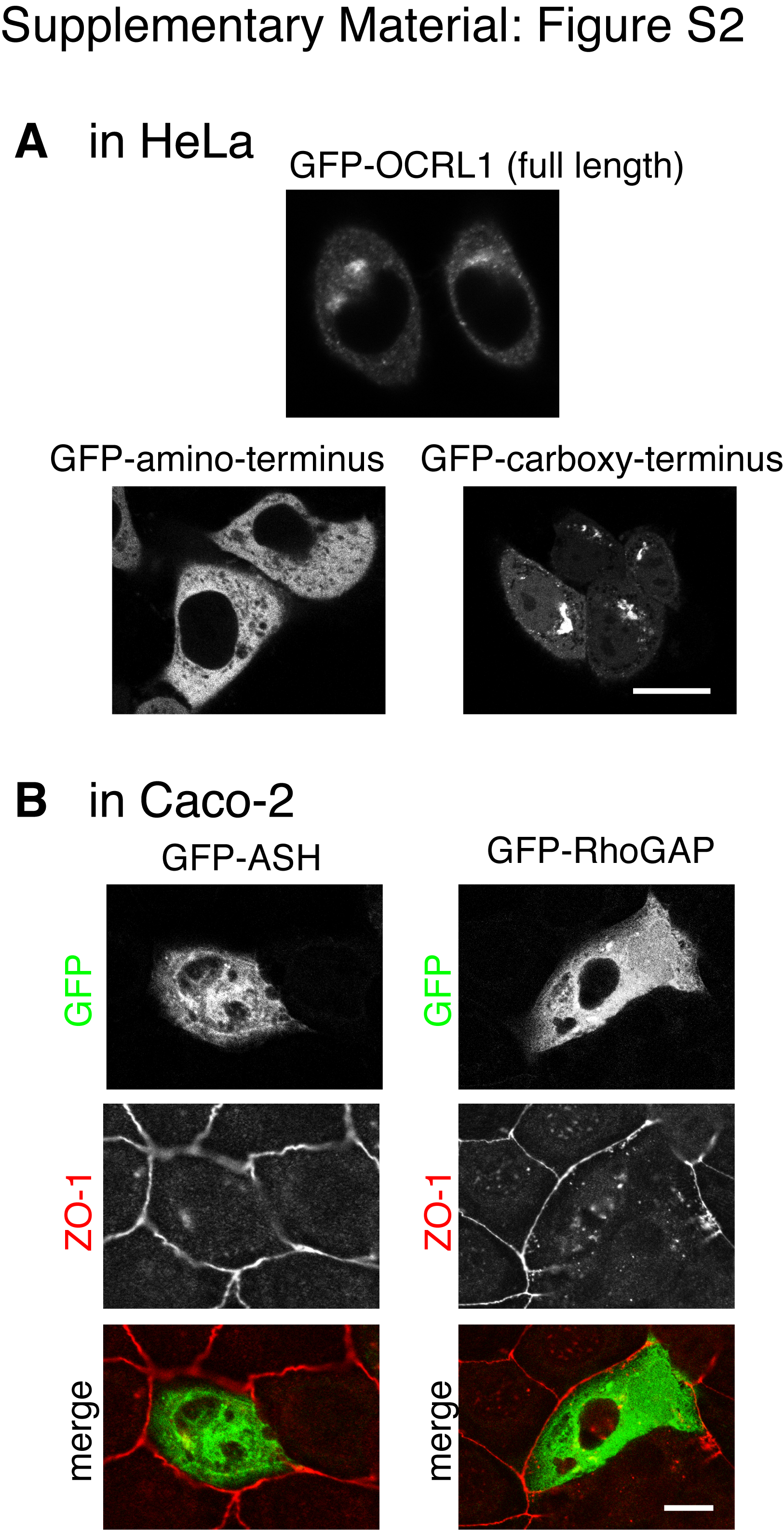

Supplement: Figure S2 — The ASH domain or the Rho-GAP domain of OCRL1 alone does not mediate junctional localization. (A) GFP-OCRL1–amino-terminus and GFP-OCRL1–carboxy terminus expressed in HeLa cells, as in Figure 3. While neither targets the periphery, the carboxy-terminus targets internal membranes as seen for polarized cells (see Figure 3E). (B) GFP-tagged ASH and Rho-GAP domains were expressed in Caco-2 cells, as in Figure 3. Neither construct targets junctional regions, as identified by ZO-1 staining. Scale bars are 20 µm. (TIF) [file pone.0024044.s002.tif]

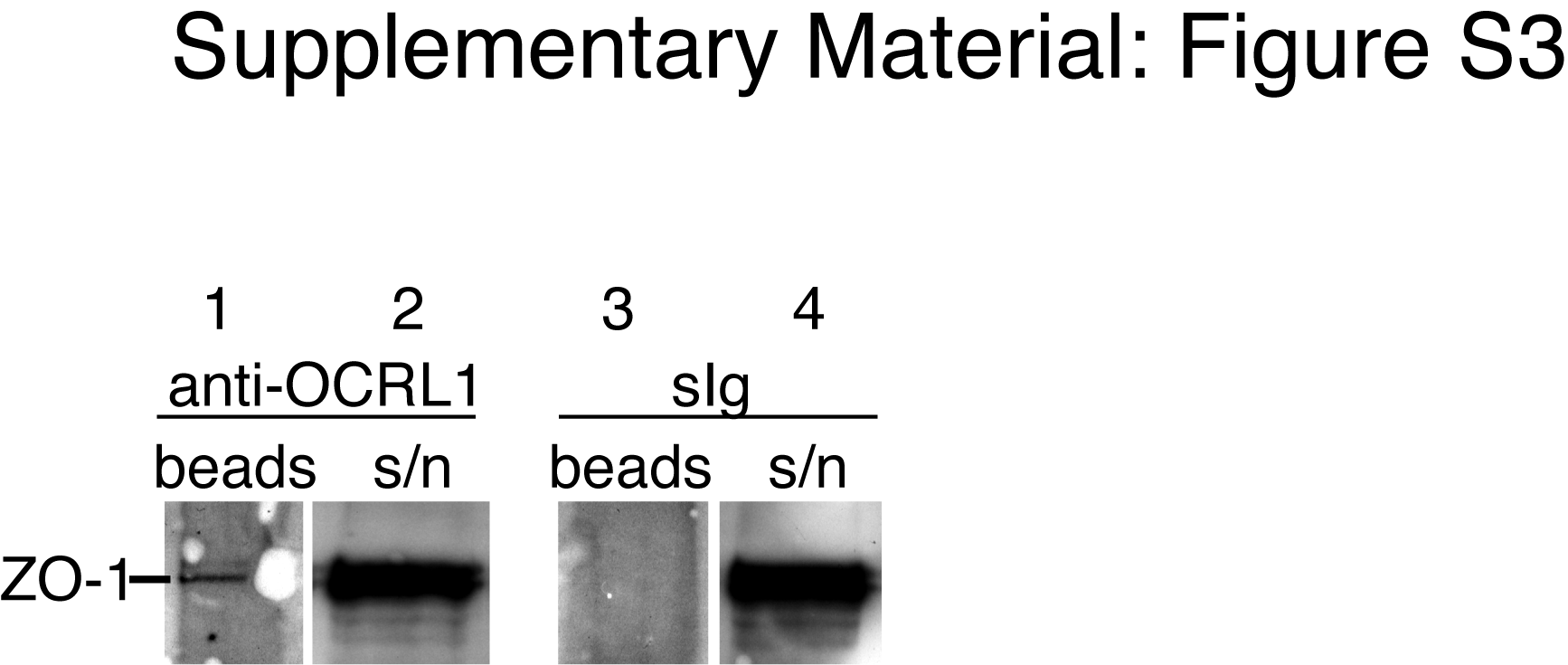

Supplement: Figure S3 — Endogenous ZO-1 immunoprecipitates with OCRL1 in Caco-2 cells. Caco-2 cell lysates were prepared as in Figure 4A. OCRL1 (lanes 1 & 2) and irrelevant antibodies (normal sheep immunoglobulin – sIg) (lanes 3 & 4) were used to precipitate proteins from a pre-cleared Caco-2 cell lysate. Precipitated proteins (30%, lanes 1 & 3) and unbound supernatants (1%, lanes 2 & 4) were separated by SDS-PAGE, and probed with antibodies to ZO-1, which runs as a doublet (arrow), only seen with anti-OCRL1. (TIF) [file pone.0024044.s003.tif]

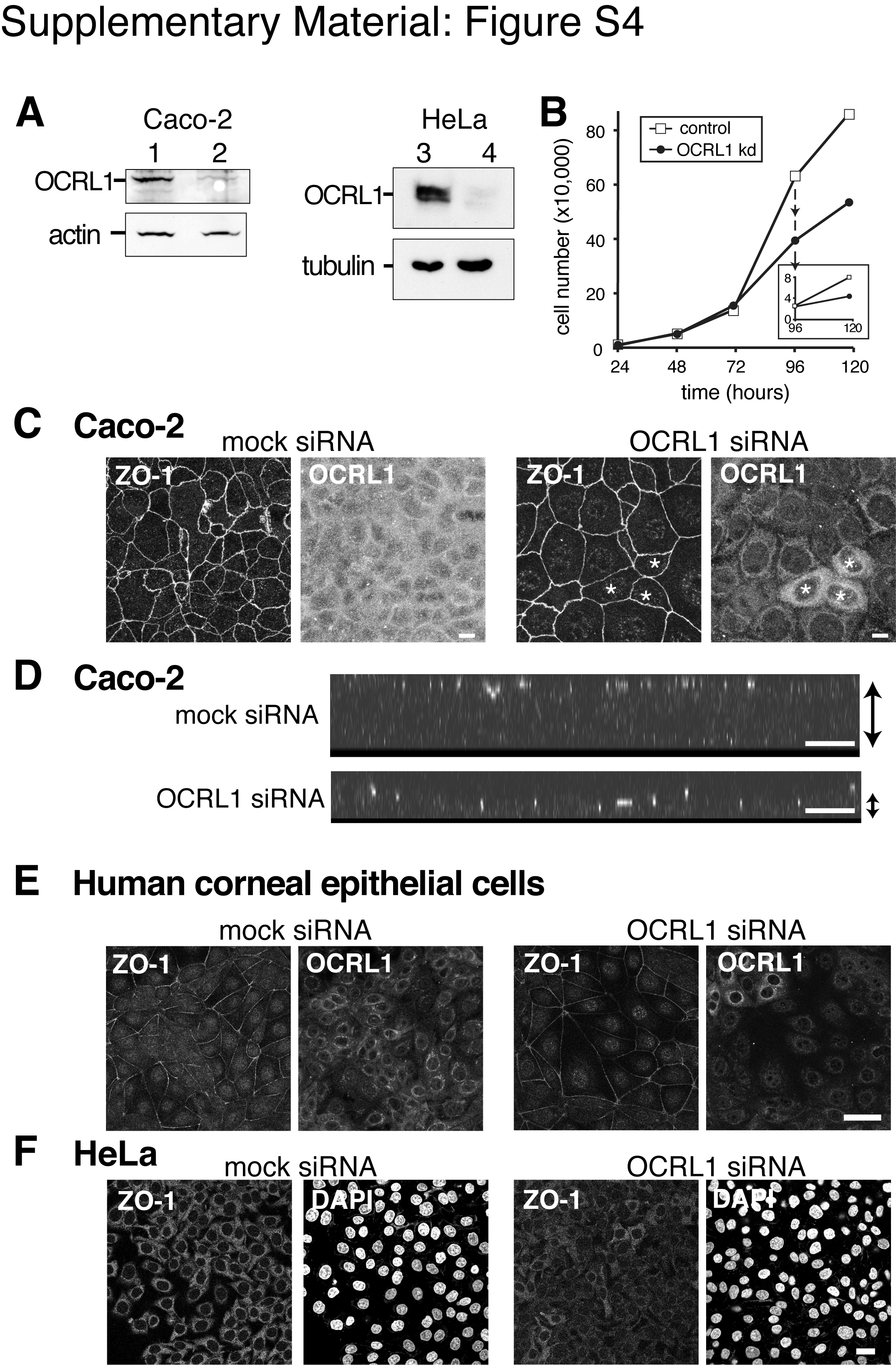

Supplement: Figure S4 — OCRL1 depletion by RNA interference in different cell lines. (A) HeLa cells were treated over 72 hours to silence OCRL1 expression and with control RNA duplexes. Immunoblot shows levels of OCRL1 compared to loading controls (actin and β-tubulin for Caco-2 and HeLa respectively). Densitometry indicated reduction by ≥85%. Similar OCRL1 depletion was obtained in Caco-2, human corneal epithelial cells, and also in MDCK cells that had been treated with any one of 4 different siRNA duplexes tested (data not shown). (B) MDCK cells were plated in 24-well plates and then treated at 24 and 48 hours of growth to silence OCRL1 expression and with control siRNA duplexes, as described in Materials and Methods. Cells were removed by trypsinization and counted from 24 hours onwards. Inset: confluent cells removed from dishes at 96 hours were re-plated at 5% confluency for a further 24 hours. (C) Caco-2 cells were treated as in (B), fixed and immunostained as in Figure 1. Images are compressed confocal stacks. ZO-1 staining delineates cell borders. The increase in cross-sectional area for cells depleted of OCRL1 (bottom) compared to mock treated cells (top) was 2.4-fold (s.e.m. = 0.4, p<10−18) in multiple fields of cells taken from two separate experiments. Note that these cells have been growing to >70% confluence over 96 hours, so the absence of junctional OCRL1 is similar to that seen in Figure 1F. Asterisks indicate 3 cells unaffected by OCRL1 siRNA. (D) XZ sections of ZO-1-stained cells from (B). The mean cell heights in these XZ sections (arrows at right-hand side) are: control = 13.5 µm, OCRL1-slienced = 5 µm, and over multiple fields the average height in cells depleted of OCRL1 = 55% (±7%, p<0.001) of control. Note these cells are taller than those in Figure 5B, mainly because they were growing at a greater confluency. (E) Human corneal epithelial cells were treated to silence OCRL1 expression, and immunostained for OCRL1 (as in B), which shows reduced expression except in [file pone.0024044.s004.tif]

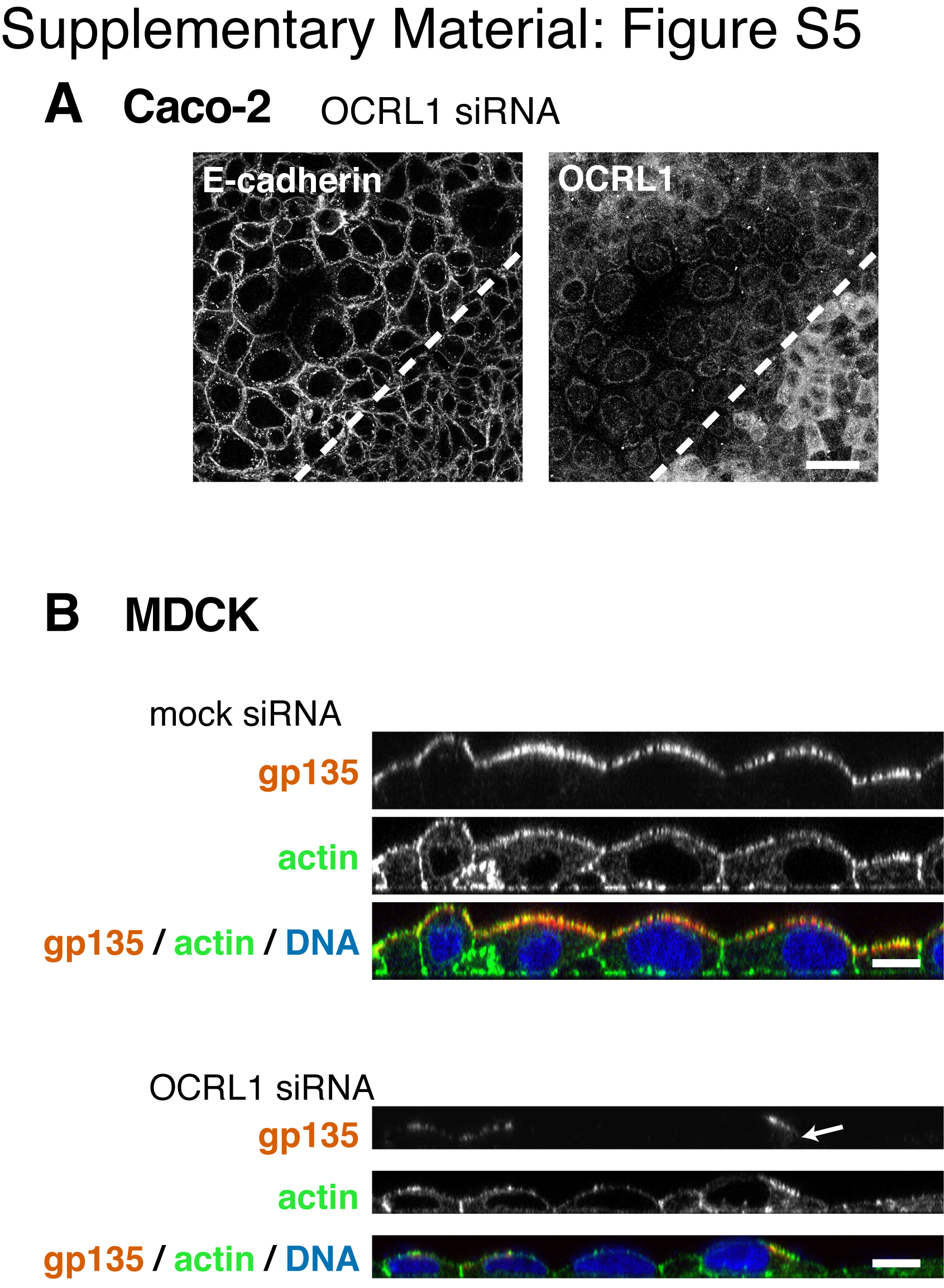

Supplement: Figure S5 — Effect of OCRL1 knock-down on E-cadherin and on gp135. (A) Caco-2 cells treated to silence OCRL1 (as in Figure S4C) were fixed and stained with anti-E-cadherin antibodies. As an internal control, cells in the lower right-hand corner (indicated by dashed line) resisted the knock-down of OCRL1, and had the same tall/columnar shape as wild-type cells (data not show). A similar pattern of E-cadherin at junctions and internal puncta is seen both in large, flat cells lacking OCRL1 and taller OCRL1+ve cells, although in the latter cells with far less cross-sectional area the puncta are crowded against the periphery. Scale bar 20 µm. (B) MDCK cells treated to silence OCRL1 were stained for the actin, DAPI and the apical marker gp135/podocalyxin. Experiment is similar to Figure 5A, but cells were fixed 24 hours earlier (i.e. 48 hours after initial siRNA treatment), explaining flatter shape. In control cells, actin and gp135 are enriched continuously across the apical domain,. In cells lacking OCRL1 there is failure of actin accumulation in the apical domain, and gp135 is either not expressed, or poorly expressed in just one segment of the apical domain, with some accumulation in a more basal region (arrow). Scale bars 10 µm. (TIF) [file pone.0024044.s005.tif]
